# Dedifferentiation and metabolic reprogramming of human adipocytes in the tumor niche triggered by colorectal cancer cells

**DOI:** 10.1186/s40659-025-00660-z

**Published:** 2025-12-06

**Authors:** Katarzyna Pietraszek-Gremplewicz, Joanna Olszańska, Mikołaj Domagalski, Agata Tymińska, Aneta Skoniecka, Michał Pikuła, Dorota Nowak

**Affiliations:** 1 Department of Cell Pathology, Faculty of Biotechnology, University of Wroclaw, Joliot-Curie 14a, 50-383 Wroclaw, Poland; 2 Laboratory of Tissue Engineering and Regenerative Medicine, Division of Embryology, Medical University of Gdansk, Debinki 1, 80-211 Gdansk, Poland

**Keywords:** Cancer-associated adipocytes, Colorectal cancer (CRC), Tumor microenvironment (TME), Adipocytes, Metabolic reprogramming

## Abstract

**Background:**

Tumor development and formation are primarily influenced by the interactions of surrounding tissues, and the cells of these become incorporated into the vicinity of the tumor and shape its microenvironment. In particular, adipose tissue plays a pivotal role, and its primary cellular components, adipocytes, make a significant contribution to this process. The multifaceted role of fat cells in the formation and progression of cancer remains an active area of research, and many aspects of this process remain undefined. Thus, the main objective of this study was to investigate how colorectal cancer (CRC) cells influence human-derived adipocytes reprogramming.

**Results:**

Our results demonstrate that CRC cells promote the dedifferentiation of adipocytes into a more fibroblast-like phenotype, and this process resulted in the formation of cells with characteristics resembling those of cancer-associated adipocytes (CAAs). Furthermore, co-culture with cancer cells disrupted cytoskeletal homeostasis in adipocytes, which enhanced the formation of actin filaments and led to the development of a more complex vimentin network. This was accompanied by alterations in lipid droplet profiles and the levels of proteins involved in lipid storage and metabolism. Interestingly, CRC cells also modulated the metabolic activity of CAAs and affected their mitochondrial distribution and dynamics.

**Conclusions:**

The results underscore the substantial influence of CRC cells on adipocytes, which may have an essential role in their recruitment into the tumor niche and protumorigenic activity.

**Graphical abstract:**

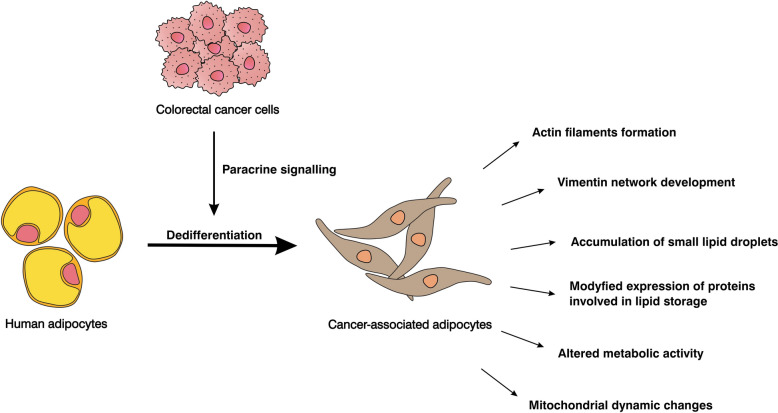

## Background

Adipose tissue is predominantly composed of adipocytes accompanied by other types of cells, such as pericytes, endothelial cells, immune cells, and pluripotent stem cells. The role of adipose tissue was formerly believed to be limited to energy storage and thermal protection [1].Further research has pointed to adipocytes being a source of a wide array of effectors, including exosomes, miRNA, lipids, and bioactive molecules called adipokines, which may act not only as local paracrine signaling cytokines, but also at distant levels through secretion in the circulation and inducing systemic metabolic responses [2]. Under physiological conditions, adipocytes regulate numerous processes connected with appetite and energy balance, such as lipid metabolism, glucose homeostasis, insulin sensitivity, angiogenesis, blood pressure, and inflammatory processes [3, 4]. Moreover, adipocytes constitute a considerable portion of the tumor microenvironment (TME) and are present in the stroma of breast, colorectal, and endometrial tumors [5]. Although immune cells and fibroblasts have been broadly researched, studies on adipocytes as primary components of the TME are only emerging.

Adipocytes in the region of a tumor dynamically exchange signals with cancer cells, which leads to morphological and functional transformations. Such adipocytes have been termed cancer-associated adipocytes (CAAs), which exhibit phenotypic and functional alterations as a consequence of their proximity to invasive cancer cells. In comparison to mature naïve adipocytes, CAAs show morphological changes that include a loss of lipid content [6, 7]. CAAs are also characterized by decreased expression of adipocyte-differentiation markers, as well as increased production of inflammatory cytokines, growth, and angiogenic factors [8]. These cells secrete collagen and matrix metalloproteases (MMPs), which remodel the extracellular matrix and promote tumor-cell invasion [9, 10]. CAAs undergo metabolic adaptation in a nutrient-restrictive environment, and their metabolism is switched toward catabolic processes, leading to the release of high-energy metabolites that can be consumed by cancer cells [5]. Cross-talk between adipocytes and cancer cells is postulated to actively contribute to the initiation and progression of tumors. Thus, comprehensive characterization of CAAs is essential to understand the development of the malignant TME.

Our previous research examined the loss of lipid content observed in adipocytes derived from the mouse 3T3-L1 cell line upon exposure to CRC cells. The results demonstrated that this loss may be a consequence of the inhibition of adipogenesis, increased lipolysis, and reduced expression of genes involved in lipid metabolism [11]. In the present study, we aimed to determine and characterize the phenotypic and metabolic alterations in human adipocytes provoked by CRC cells. We used primary human adipocytes to achieve this goal as they better reflect the TME conditions and interactions than the cells used in the previous study. The fat cells were co-cultured with CRC cells to generate CAAs, and their functional and molecular differences were characterized and compared between control (naïve) adipocytes and those reprogrammed by cancer cells.

## Methods

### Isolation and culture of adipose tissue-derived mesenchymal stromal cells (AD-MSCs)

Human subcutaneous adipose tissue was obtained from donors at a plastic surgery clinic (Gdansk, Poland). All procedures were performed after informed consent was obtained from patients and permission was granted by the Bioethics Committee for Scientific Research of the Medical University of Gdansk (NKBBN/858/2022–2023). The isolation was based on previously described protocols [12–14]. All cells were grown at 37 °C in 5% CO_2_ in a humidified atmosphere.

### Adipocyte differentiation and co-culture with CRC cells

Human adipocytes were obtained from AD-MSCs according to a previously described protocol [14, 15]. For CAA induction, differentiated adipocytes were co-cultured with CRC cells (LS180 (Deutsche Krebsforschungzentrum, Heidelberg, Germany), HCT116, or LoVo (European Collection of Cell Cultures)), which were seeded onto a Transwell insert (0.4-μm pores, Falcon). Cells were grown for 7 days in DMEM/F12 medium (Gibco) supplemented with 10% FBS (Gibco), 2 mM glutamine (Sigma-Aldrich), and antibiotic–antimycotic solution (100 U/ml penicillin, 100 µg/ml streptomycin, 0.25 µg/ml amphotericin B) (Gibco). The medium was refreshed every 3–4 days. A culture of mature adipocytes was run in parallel as a control. After co-culture, CAAs were collected and used for further experiments. All experiments were performed with cells that had been isolated from at least three different donors.

### qRT-PCR analysis

To assess the expression levels of different genes, RNA was isolated from cells using Fenozol reagent (A&A Biotechnology) according to the manufacturer’s instructions. After on-column digestion with DNase I (EURx), RNA was used for reverse transcription reaction with a High-Capacity cDNA Reverse Transcription Kit (Applied Biosystems) according to the manufacturer’s protocol. Quantitative PCR was performed using PowerUp™ SYBR™ Green Master Mix or TaqMan™ Fast Universal PCR Master Mix (Thermo Fisher Scientific). All results were normalized to the expression level of the hypoxanthine phosphoribosyltransferase 1 (*HPRT1*) gene based on the ΔΔCT method. The following TaqMan probes were used: HPRT1: Hs03929098_m1, LEP (Leptin): Hs00174877_m1, AdipoQ (adiponectin): HS00605917_m1, NAMPT (nicotinamide phosphoribosyltransferase/ visfatin): Hs00237184_m1 (Thermo Fisher Scientific). All used primers were purchased from Merck, and their sequences are listed in Table 1.

**Table 1:** Sequence of used primers

Abbreviation	Full name of the gene	Forward primer 5’-3’	Reverse primer 5’-3’
*HPRT1*	hypoxanthine phosphoribosyltransferase 1	GACCAGTCAACAGGGGACAT	GCTTGCGACCTTGACCATCT
*NES*	nestin	AGCCCTGACCACTCCAGTTTAG	AGTCCTGGATTTCCTTCCTGTTTAGAT
*ITGB1*	integrin β1	TGAATGCCAAATGGGACACG	TGACCACAGTTGTTACGGCA
*CD90*	cluster of differentiation 90	GCAGAAGGTGACCAGCCTAAC	AAGTTGGTTCGGGAGCGGTA
*ACTA2*	actin alpha 2, smooth muscle	CTTCCATTGTCCACCGCAAAT	AGAGCTTTGGCTAGGAATGATTTG
*FAP-1*	fibroblast activation protein 1	GCCTTATTGGTGATGTGCAT	TCTTGTCCTGAAATCCAGTTTG
*PLIN1*	perilipin1	GAGCTGAAGGACACCATCTC	GTACTCCACCACCTTCTCAATG
*PPARγ*	peroxisome proliferator-activated receptor γ	CCATTCTGGCCCACCAACTTTG	GGTCATACTTGTAATCTGCAACCACT
*LDLR*	low-density lipoprotein receptor	GACCGTCGCCTTGCTCCTCGC	CCGGATTTGCAGGTGACAGACAAG
*CIDEC*	cell death-inducing DFFA-like effector C	CATGTGTCAGTGCGTACCTCT	GATGCCCTTCCTCACGCTTC
*FABP4*	fatty acid binding protein 4	GATTATATGAAAGAAGTAGGAGTGGGCTT	CCATCTAAGGTTATGGTGCTCTTGAC
*FABP5*	fatty acid binding protein 5	GCAGCTGGAAGGAAGATGGC	AACTTCTCTCCCAGGGTACAAGAA
*CD36*	cluster of differentiation 36 / fatty acid translocase	CTAATGCCAGTTGGAGACCTGC	GCTGCTGTTCATCATCACTTCCT
*FATP1*	long-chain fatty acid transport protein 1	TGCTGCAGCTCCATGTGAC	CTGACAGTGGTGACATCCAAGT
*VIM*	vimentin	GCTTCGCCAACTACATCGACAAG	CTTTGTCGTTGGTTAGCTGGTCC

### Western blotting analysis

Cells were lysed with urea buffer (50 mM Tris, pH 7.4, 5% SDS, 8.6% sucrose, 74 mM urea, 1 mM dichlorodiphenyltrichloroethane), which was supplemented with protease and phosphatase inhibitor cocktails (Sigma Aldrich). The protein concentration in samples was evaluated using a standard bicinchoninic acid (BCA) method (Thermo Fisher). Samples with the same amounts of protein were separated by SDS-PAGE as reported by Laemmli [16] and then transferred to nitrocellulose membranes as reported by Towbin et al. [17]. We employed primary antibodies against perilipin1 (Cell Signaling; 9349T), CD36 (Abcam; ab133625), RACK1 (Santa Cruz Biotechnology; sc-17754), β catenin (Cell Signaling Technologies; 9562), MIEF1 (Proteintech; 20,164–1-AP), and OPA1 (Santa Cruz Biotechnology; sc-393296), as well as goat anti-rabbit and anti-mouse secondary antibodies conjugated with horseradish peroxidase (Cell Signaling). Detection was performed with Clarity Western ECL Substrate (Bio-Rad) or Clarity Max Western ECL Substrate (Bio-Rad, Bio-Rad) under ChemiDoc (Bio-Rad) and evaluated with ImageLab software (ver. 6.0, Bio-Rad). All results were normalized to the total protein content evaluated with Ponceau S staining [18, 19].

### Antibody arrays

The molecules secreted by CAAs were analyzed using a Human Adipokine Antibody Array and Proteome Profiler Human Angiogenesis Array (R and D Systems). After co-culture, cells were washed three times with PBS, and the culture media were changed to fresh media without FBS for another 72 h. Next, the conditioned media were collected, and arrays were performed according to the manufacturer’s protocol. The signal was detected using streptavidin-HRP. The chemiluminescence signal was measured using a ChemiDoc Imaging System (Bio-Rad) and analyzed with ImageLab software (Bio-Rad). Densitometric values were background corrected and then normalized to the mean of reference spots for each membrane.

### Immunocytochemistry

The subcellular distributions of cell nuclei, lipid droplets (LDs), F-actin, G-actin, mitochondria, and vimentin were examined using fluorescence confocal microscopy. Cells cultured on coverslips were fixed with 4% formaldehyde and permeabilized with 0.1% Triton X-100 in PBS. Lipid droplets were detected by LipidSpot 488 (Biotium), mitochondria were detected with MitoTracker® Orange CMTMRos (Invitrogen), and nuclei were detected with Hoechst 33,342 (Invitrogen). Actin filaments were detected with Phalloidin CruzFluor™ 488 Conjugate (Santa Cruz Biotechnology), and G-actin was detected with Alexa Fluor™ 594-labeled DNase I (ThermoFisher Scientific). Cytoplasm was stained with high-content screening CellMask™ Deep Red Stain (Invitrogen). Rabbit anti‐vimentin antibody (GeneTex) followed by Alexa Fluor 488‐conjugated anti‐rabbit secondary antibody (Invitrogen) was used to detect this protein.

Confocal images for quantitative measurements were acquired using an Opera Phenix Plus System (Perkin Elmer), and the results were calculated by dedicated Harmony software. Data were obtained from at least 500 cells for each condition and repetition. The level of vimentin protein was estimated as the sum of the intensity of the vimentin-positive pixels and normalized to the number of nuclei present in the image area. Similarly, the ratio of F- to G-actin was calculated based on the quantification of fluorescence intensity of F- and G-actin and then normalized to the number of nuclei. For more precise analysis of subcellular mitochondria, F and G-actin, and the vimentin distributions, confocal images were captured using a Leica Stellaris 8 (Leica, Wetzlar, Germany) and LAS X software (ver. 3.3.0, Leica, Wetzlar, Germany). Representative areas are shown for each condition.

### ATP (adenosine triphosphate) production rate and evaluation of mitochondrial respiration

Cell metabolic status was investigated using a Seahorse XF Pro extracellular flux analyzer (Agilent Technologies) with 96-well Seahorse microplates. A Seahorse XF Real-Time ATP Rate Assay kit and Cell Mito Stress Test Kit (Agilent Technologies) were applied according to the manufacturer’s instructions. The oxygen consumption rate (OCR) and the extracellular acidification rate (ECAR) were measured. The assays were done in non-buffered DMEM containing 10 mM glucose, 2 mM glutamine, and 1 mM pyruvate (Agilent Technologies). For the ATP production-rate assay, the injection sequence was oligomycin (1 μM at final concentration), followed by rotenone and antimycin A (0.5 μM at final concentrations). For the Cell Mito Stress Test, the order of injections was oligomycin (1.5 μM at final concentration), followed by FCCP (carbonyl cyanide-4 (trifluoromethoxy) phenylhydrazone, 1 μM at final concentration), and then rotenone and antimycin A (0.5 μM at final concentrations). At the end of the Seahorse measurements, cell nuclei were stained with Hoechst 33,342 (Invitrogen), imaged, and counted using Citation 5 (Agilent BioTek). The results were directly used to normalize the Seahorse parameters according to the number of cells. The data were calculated by Seahorse Analytics (Version: 1.0.0–720, Agilent Technologies) and are presented as percentages of the control result.

### Statistical analysis

All data are shown as the mean ± standard deviation (SD). Significance was determined using GraphPad Prism 7 software and the Kruskal–Wallis test with Dunn’s multiple-comparisons test. The criteria for significance were set as *p* < 0.05 (*), *p* < 0.01 (**), and *p* < 0.001 (***).

## Results

**Fig. 1:**
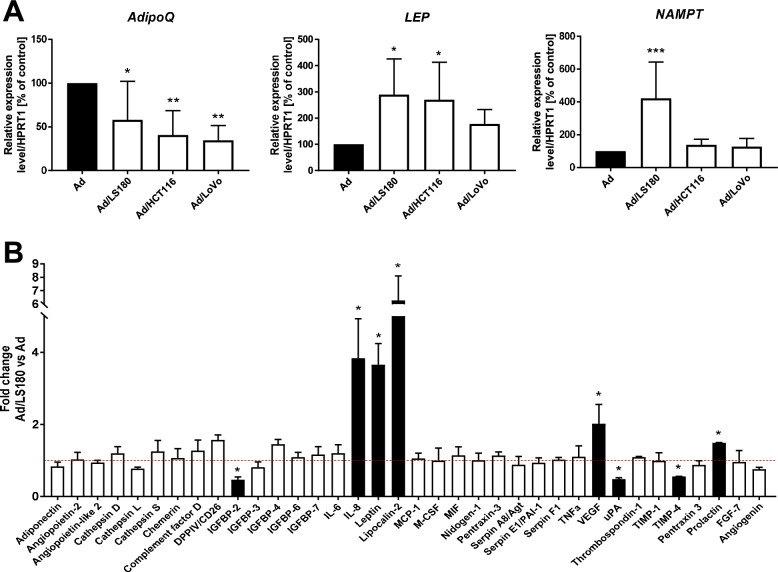
Level of adipokines in CAAs. **A** qRT-PCR analysis of *AdipoQ* (*adiponectin*, n = 6),* LEP* (*leptin*, n = 5), and *NAMPT* (*visfatin*, n = 6) expression by control adipocytes (Ad) and adipocytes co-cultured with CRC cells (Ad/LS180, Ad/HCT116, and Ad/LoVo). mRNA level was normalized against the expression of *HPRT1* (*hypoxanthine* *phosphoribosyltransferase 1*). **B** Adipokines secreted into culture media were detected by antibody arrays. Based on the obtained signals, quantitative analysis was conducted with normalization to the mean of reference spots, and the result was calculated as the fold change Ad/LS180 vs. Ad (n = 3). The results from at least three biological replicates are presented as the mean ± SD. The significance level was set at *p* ≤ 0.05 (*), *p* ≤ 0.01 (**), and *p* ≤ 0.001 (***). Abbreviations: DPPIV: dipeptidyl peptidase 4; IGFBP-2, -3, -4, -6, -7: insulin-like growth factor-binding protein-2, -3, -4, -6, -7; IL-6, -8: interleukin-6, -8; MCP-1: monocyte chemoattractant protein-1; M-CSF: macrophage colony-stimulating factor; MIF: macrophage migration inhibitory factor; Agt: angiotensinogen; PAI-1: plasminogen activator inhibitor-1; TIMP-1, -4: tissue inhibitor of metalloproteinases-1, -4; FGF-7: fibroblast growth factor 7; TNFa: tumor necrosis factor-alpha; VEGF: vascular endothelial growth factor; uPA: urokinase-type plasminogen activator

To obtain CAAs, differentiated adipocytes were co-cultured with 3 different CRC cell lines (LS180, HCT116, and LoVo). Next, we assessed the transformation of adipocytes (Ad) into CAAs (Ad/LS180, Ad/HCT116, or Ad/LoVo) based on typical markers for differentiated adipocytes, such as adiponectin, leptin, and visfatin (NAMPT, nicotinamide phosphoribosyltransferase). The mRNA expression of *adiponectin (AdipoQ)* in the CAAs was decreased, while there was increased expression of *leptin (Lep)* and *NAMPT* (the latter only for Ad/LS180) (Fig. 1A). In light of the fact that fat cells are the prominent source of biologically active molecules, this result prompted us to broaden the analysis with the use of LS180 cells, which in previous experiments seemed to impact adipocyte transformation in a more prominent way than the two other cell lines. Antibody arrays revealed modifications in the adipokines’ secretion profile of CAAs induced by LS180 cells (Fig. 1B). Among the multiple molecules for which secretion was detected, significant upregulation was noted for interleukin-8 (IL-8), leptin, lipocalin-2, vascular endothelial growth factor (VEGF), and prolactin, which are all considered to be pro-inflammatory [20–24] and pro-angiogenic factors [25–29].

**Fig. 2:**
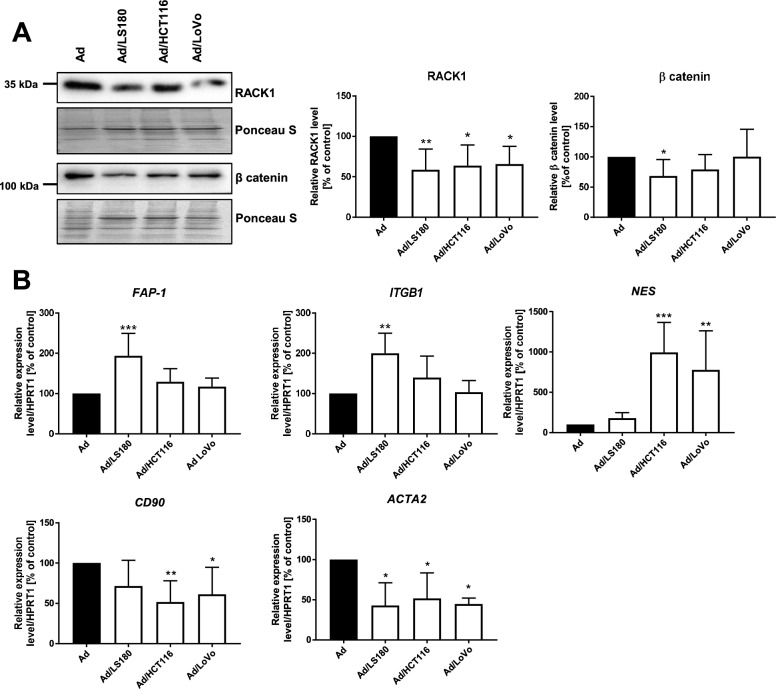
Characterization of CAAs. **A** Western blotting analysis of RACK1 (receptor for activated C kinase 1, n = 6) and β catein (n = 5) level normalized to total protein level (Ponceau S staining). **B** mRNA expression level of *FAP-1* (*fibroblast activation protein 1*, n = 7), *ITGB1* (*integrin β1*, n = 6), *NES* (*nestin*, n = 6), *CD90* (n = 7), and *ACTA2* (*actin alpha 2*, *smooth muscle*, n = 3) normalized against the expression of *HPRT1* (*hypoxanthine phosphoribosyltransferase 1*). The results from at least three biological replicates were normalized to the control and are presented as the mean ± SD. The statistical significance is indicated by asterisks and set at *p* ≤ 0.05 (*), *p* ≤ 0.01 (**), and *p* ≤ 0.001 (***)

The altered levels of adipokines observed in the microenvironment of CAAs may indicate activation and dedifferentiation of these cells. The receptor for activated C kinase 1 (RACK1) is required for adipocyte differentiation by emerging Wnt/β-catenin signaling [30]. After exposure of adipocytes to CRC cells, there were decreases in the protein levels of both RACK1 and β-catenin (although the reduction for β-catenin was significant only in the case of Ad/LS180) (Fig. 2A). It is postulated that adipocytes may display features of fibroblasts under the influence of cancer cells [5]. We identified upregulated expression of *fibroblast activation protein-1 (FAP-1)*, *integrin β1 (ITGB1)* (significant only in Ad/LS180 cells), and *nestin* (*NES*) (Fig. 2B) in adipocytes upon co-culture. Induced CAAs exhibited reduced levels of *CD90* and *actin alpha 2, smooth muscle* (Fig. 2B).

**Fig. 3:**
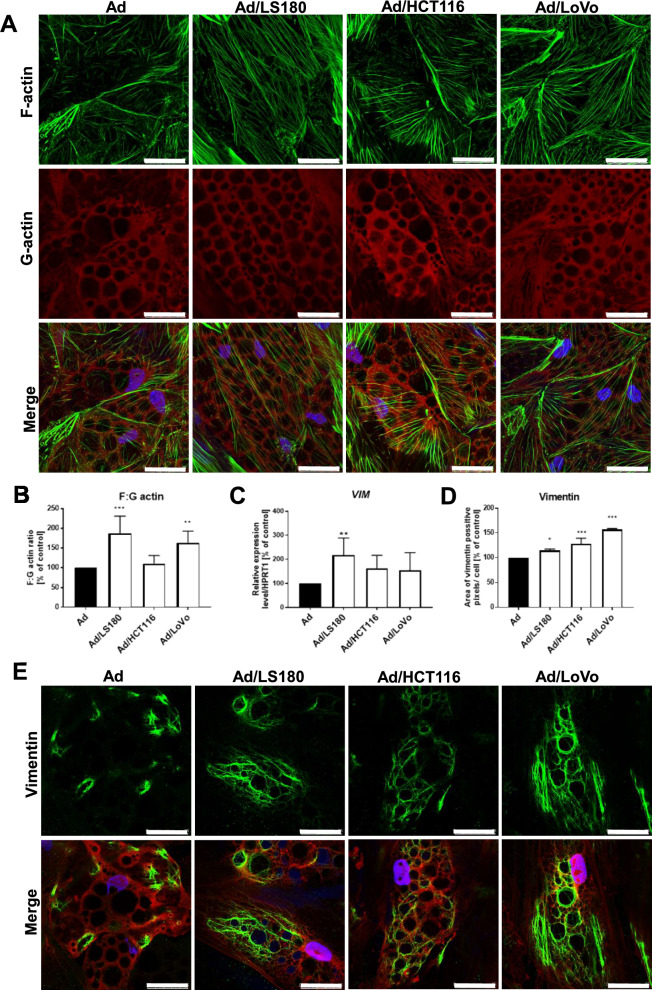
Rearrangement of adipocytes' cytoskeleton induced by CRC cells. **A** Representative images of control adipocytes (Ad) and adipocytes co-cultured with CRC cells (Ad/LS180, Ad/HCT116, Ad/LoVo) stained for F-actin (green), G-actin (red), and cell nuclei (blue) with **B** calculation from high-content screening microscopy of F:G actin ratio based on quantification of fluorescence intensity (n = 6). **C** qRT-PCR analysis of *vimentin* (*VIM*, n = 7) expression normalized to *HPRT1* expression. **D** High-content screening confocal microscope quantification of pixels corresponding to vimentin expression (n = 4). **E** Representative image of cellular distribution of vimentin (green), cell nuclei (blue), and cytoplasm (Cell Mask, red). Scale bar: 25 µm. Results are presented as the mean from at least three independent experiments ± SD and were calculated as a percentage of the control result. The significance level was set at *p* ≤ 0.05 (*), * p* ≤ 0.01 (**), and *p* ≤ 0.001 (***)

Analysis of the CAAs’ actin cytoskeleton organization also indicated their similarity to fibroblasts, which were characterized by a spindle-shaped morphology and a large number of stress fibers [31]. Analogously, confocal microscopy images showed that the acquired CAAs had a longer and more developed filamentous (F)-actin network, which indicated an increase in stress fiber alignment (Fig. 3A). This observation was confirmed by fluorescence quantification showing a greater F:G-actin ratio in these cells (Fig. 3B). Vimentin, the only intermediate filament protein expressed in preadipocytes regulating adipogenesis [32], is also a characteristic protein for fibroblasts. Adipocytes co-cultured with CRC cells showed increases in the levels of both vimentin mRNA (significant only in Ad/LS180 cells, Fig. 3C) and vimentin protein (Fig. 3D). Moreover, the network formed by vimentin fibers was more developed in CAAs, and the long vimentin filaments covered the entire cytoplasm of the cells (Fig. 3E).

**Fig. 4:**
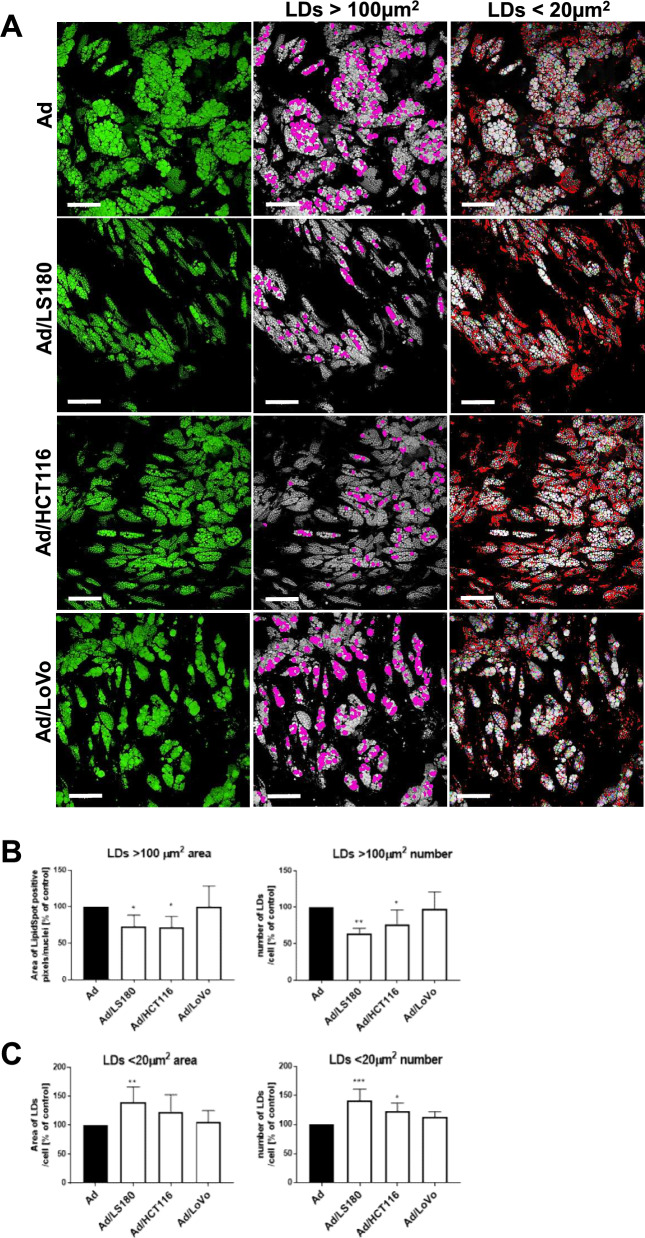
Distribution of LDs in CAAs. **A** Representative images of control adipocytes and adipocytes incubated with CRC cells stained for neutral lipids with Lipid Spot 488 (marked in green) with an illustration of the LD division into fractions based on their area: > 100 µm^2^ (marked in magenta), and < 20 µm^2^ (marked in red). Scale bar: 200 µm. Quantification of area and number of LDs: **B** > 100 µm^2^ and **C** < 20 µm^2^. Graphs present the mean ± SD of results in comparison to the control (n = 7). Asterisks indicate statistically significant differences at the level of *p* ≤ 0.05 (*), *p* ≤ 0.01 (**), and *p* ≤ 0.001 (***)

A fibroblast-like phenotype of adipocytes in the TME was previously described as an adaptation due to dispersed LDs [33]. We also observed a decrease in both the area and number of large LDs (> 100 µm^2^) in the tested CAAs compared with the control adipocytes (Fig. 4A, B). At the same time, adipocytes treated with CRC cells accumulated a higher amount of small vimentin (< 20 µm^2^) (Fig. 4A, C). We did not detect any significant differences in the number or total area of intermediate 20–100-µm^2^ LDs (data not shown).

**Fig. 5:**
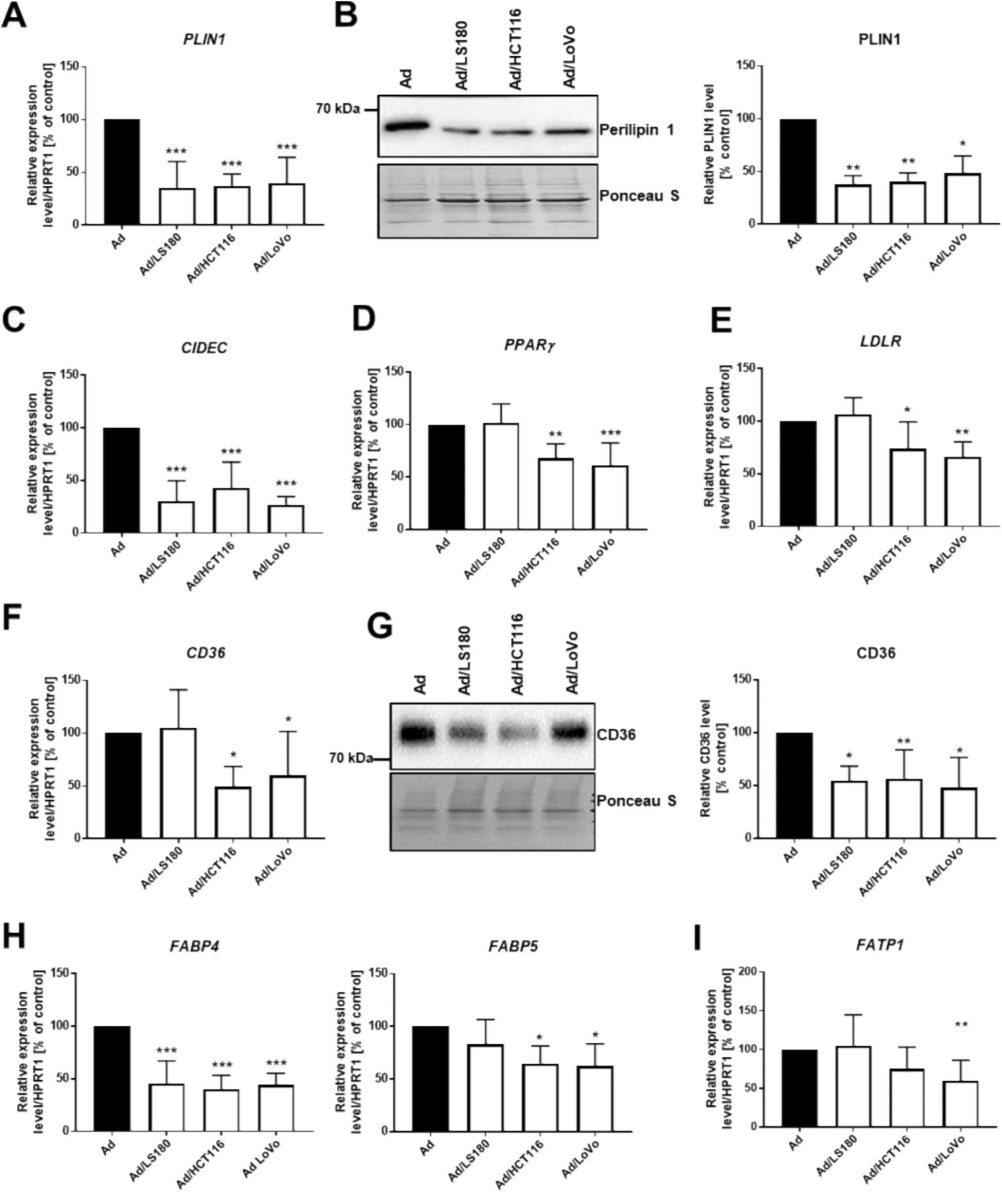
Expression level of proteins involved in lipid storage in adipocytes co-cultured with CRC cells. mRNA level of **A ***perlipin1* (*PLIN1*, n = 5), **C ***cell death-inducing DFFA-like effector C* (*CIDEC*, n = 4), **D ***peroxisome proliferator-activated receptor γ* (*PPAR*ɣ, n = 6), E low-density lipoprotein receptor (LDLR, n = 6), F CD36 (n = 7), H fatty acid binding protein 4 and 5 (FABP4, FABP5, n = 4), and I long-chain fatty acid transport protein 1 (FATP1, n = 7) in control (Ad) and cancer-associated adipocytes (Ad/LS180, Ad/HCT116, and Ad/LoVo) assessed by qRT-PCR normalized against the expression of HPRT1. Protein level of B PLIN1 (n = 6)and G CD36 (n = 6) analyzed by western blotting technique normalized to the total protein level (Ponceau S staining). The results from at least three biological replicates were normalized to the control and are presented as the mean ± SD. The significance level indicated by asterisks was set at p ≤ 0.05 (*), p ≤ 0.01 (**), and p ≤ 0.001 (***)

CAAs also had lower expression of perlipin1 (Fig. 5A, B) and mRNA of *cell death-inducing DFFA-like effector C* (*CIDEC*, Fig. 5C) in comparison to the control adipocytes. Both of these are involved in LD formation and maintenance [34]. Peroxisome proliferator-activated receptor γ (PPARγ) is the key transcriptional regulator of adipogenesis and directly triggers many genes involved in adipocyte lipid storage [35], including *perilipin 1* (*PLIN1*) [36]. Since the expression of this transcriptional factor was decreased upon exposure of adipocytes to CRC cells (Fig. 5D), we investigated the expression level of other proteins involved in lipid uptake and storage in adipocytes. The analysis showed that CAAs expressed lower levels of mRNA for *low-density lipoprotein receptor* (*LDLR*, Fig. 5E) and CD36 (at mRNA (Fig. 5F) and protein (Fig. 5G) levels). The same cells exhibited lower expression of *fatty acid binding protein 4* and* 5* (*FABP4* and *FABP5*, Fig. 5H) and *long-chain fatty acid transport protein 1* (*FATP1*, Fig. 5I), with the latter being significantly reduced only in the case of Ad/LoVo cells. These data show that CAAs may have reduced lipid uptake and storage capabilities in comparison to the control adipocytes.

**Fig. 6:**
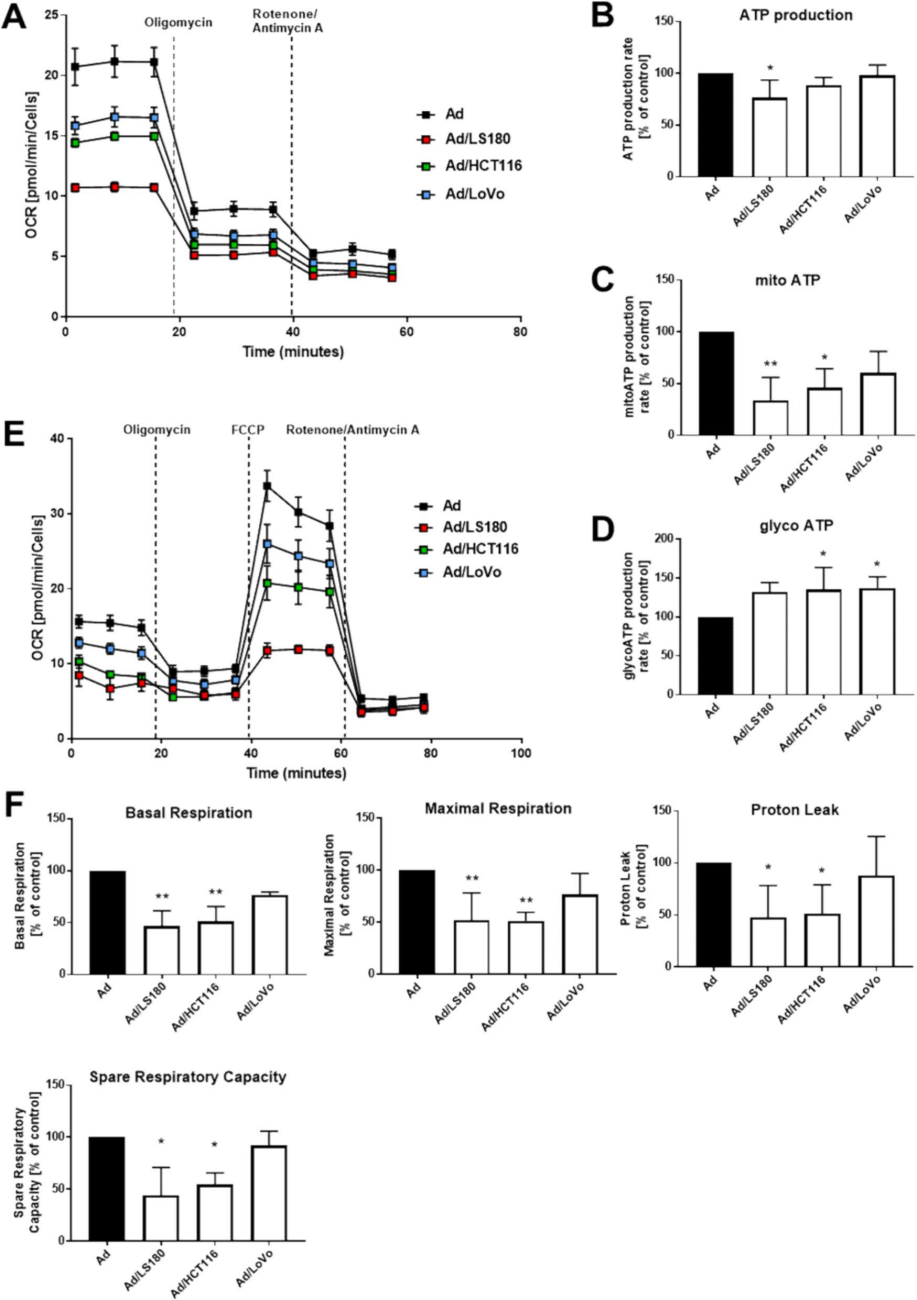
Metabolic function of adipocytes co-cultured with CRC cells. **A** Representative measurements of OCR in control cells (Ad) and CAAs (Ad/LS180, Ad/HCT116, Ad/LoVo) during ATP rate assay. **B** Total, **C** mitochondrial, and **D** glycolysis-based ATP production rate calculated based on the oxygen consumption rate (OCR) and the extracellular acidification rate (ECAR) measurements. **E** Representative measurement of mitochondrial function of adipocytes subjected to Mito Stress tests. **F** The relative levels of OCR associated with basal respiration, maximal respiration, proton leak, and spare respiratory capacity were calculated based on the measurements obtained upon the addition of different compounds. All results were acquired by a Seahorse XF Pro extracellular flux analyzer and are presented as the mean ± SD from five independent experiments, which were calculated as a percentage of the control result. The significance level was set at *p* ≤ 0.05 (*) and *p* ≤ 0.01 (**)

Since CRC cells induced changes in adipocytes in terms lipid uptake and storage, we decided to evaluate the metabolic status of CAAs by real-time ATP-production measurements (Fig. 6A). CAAs exhibited moderately lower total ATP production (Fig. 6B), while the proportion between the sources of this energy driver was shifted. Adipocytes co-cultured with CRC showed decreased mitochondrial ATP production rates (Fig. 6C) and enhanced glycolysis-based ATP-production levels (Fig. 6D) in comparison to control cells. The smaller level of ATP coming from oxidative phosphorylation (OXPHOS) led us to further evaluate the functioning of the mitochondrial respiration machinery by the Mito stress assay (Fig. 6E). We found that basal and maximal respiration, proton leak, and spare respiratory capacity were decreased in CAAs in comparison to the control (Fig. 6F). These data confirmed the impaired efficiency of the respiratory chain in mitochondria, which may explain the decreased OXPHOS -based ATP production in CAAs.

**Fig. 7:**
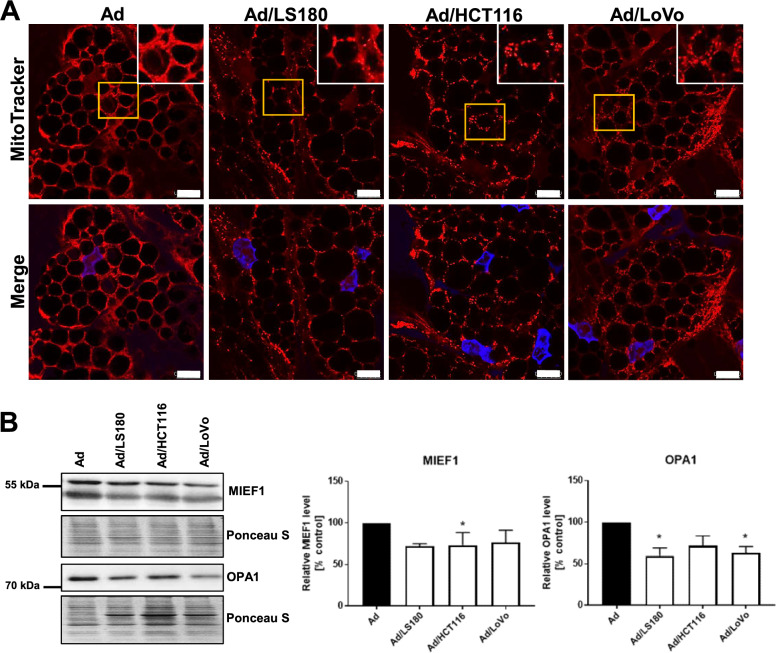
Mitochondrial changes in CAAs induced by CRC cells. **A** Representative images of cells stained for mitochondria (red) and nuclei (blue). Enlargements of the yellow boxed areas are shown as insets in the upper-right corner. Scale bar: 10 µm. **B** Western blotting analysis of mitochondrial elongation factor 1 (MIEF1, n = 4) and optic atrophy 1 (OPA1, n = 4) levels normalized to total protein content (Ponceau S staining). Representative blotting membranes are shown. The results from at least three biological replicates were normalized to the control and are presented as the mean ± SD. The significance level was set at *p* ≤ 0.05 (*)

Due to the detected metabolic differences in mitochondrial functioning between the control adipocytes and CAAs, we also evaluated the intracellular distribution of these organelles. In control adipocytes, mitochondria were evenly distributed in the spare cytoplasm volume that was not occupied by the LDs, while in CAAs, mitochondria gathered in cluster-like structures in the cytoplasm (Fig. 7A, enlarged in insets). Considering the importance of mitochondrial dynamics in cellular functions, we analyzed the levels of the mitochondrial fusion-regulating proteins: mitochondrial elongation factor 1 (MIEF1) and optic atrophy 1 (OPA1). Reduced expression of both of these proteins was detected in CAAs (Fig. 7B). Therefore, it is possible that mitochondrial fragmentation in CAAs is connected with a reduced oxidative capacity and is ultimately reflected in decreased ATP levels that are dependent on these organelles.

## Discussion

The tumor niche contains many types of cells, including CAFs, CAAs, and immune cells, and the molecules that they secrete influence the aggressiveness of cancer cells and shed light on the effectiveness of cancer treatments [37]. Apart from being a reservoir of nutrients, adipocytes also have a secretory function that affects the behavior of other cells and tissues, even distant ones. It has been demonstrated that in many types of cancer that obesity contributes to a poorer prognosis for patients [1]. Research in this area has usually focused on revealing the transformations that occur in cancer cells under the influence of adipocytes, but less interest has been given to the impact of cancer cells on adipocytes. Our previous studies clearly demonstrated mutual interactions between adipocytes derived from the mouse 3T3-L1 cell line and CRC cells [11]. In the present work, we used primary human cells derived from donors to broaden the characterization of differences between control (naïve) adipocytes and those reprogrammed by cancer cells.

CRC is a heterogeneous type of cancer and can be stratified into four consensus molecular subtypes (CMSs) according to their molecular signature, which depends in part on the composition of the tumor microenvironment (TME) and predicts clinical outcome. Specifically, CMS1 displays an immune system signature, CMS2 represents an epithelial-like cancer characterized by the activation of Wnt and c-Myc signaling pathways, CMS3 shows metabolic deregulation, and CMS4 displays a mesenchymal phenotype with remarkable upregulation of genes involved in epithelial-mesenchymal transition (EMT), matrix remodeling pathway, and transforming growth factor β (TGFβ) signaling [38]. In this study, we used CRC cell lines classified as different CMSs. LS180 cells are a type of CMS3, HCT116 are a type of CMS1, and Lovo cells represent CMS2. Recently, single-cell RNA-seq was applied to CRC samples, which revealed CMS features at the cellular level and the coexistence of multiple CMS in tumors of individual patients [39, 40]. This finding underscores the importance of in vitro analysis of CRC cell lines belonging to different CMSs, as well as the study of their differential effects on the elements of the TME.

Our results demonstrate that all cell types used were able to transform adipocytes into CAAs. However, the response of fat cells was not the same in all evaluated parameters, which may be connected with the different origins and characteristics of the cells used. These differences are reflected in the secretome of CRC cells, which differentially induces the transformation of adipocytes into CAAs. Little is known in this area, which will be a focus of a future investigation. LS180 cells were classified as the metabolic subtype, which was previously described to have upregulated metabolic activity, including glutamine, fatty acid, and lysophospholipid metabolism [41, 42]. Thus, it is possible that this type of cell due to the more prominent metabolic alterations connected with its subtype are more effective in transformation of adipocytes into CAAs. Additionally, upregulated cellular metabolism previously described as the feature of CMS3 is always connected with increased levels of metabolites secreted into culture media, which may also affect adipocytes in co-culture conditions. However, these hypotheses should be tested in future studies.

Adipocytes can support cancer progression at many levels, which mainly occurs through the secretion of adipokines that stimulate the proliferation and migration of cancer cells [43]. It has been reported that leptin levels are increased in the plasma of patients with breast cancer, which correlates with higher grade, advanced tumor stages, and aggressive subtypes. The secretion level of this adipokine is increased in CAAs [10]. Moreover, leptin promotes the proliferation of breast-cancer cells. Adipose tissue also interacts with cancer cells via the secretion of visfatin, and elevated extracellular levels of visfatin have been linked to the promotion of tumor growth and metastasis [44]. In contrast, adiponectin acts as a protective factor against tumor progression, and its secretion has been described to decrease in CAAs [10]. In adipocytes transformed by CRC cells, we also detected altered expression of these adipokines in a pro-tumorigenic manner. Notably, apart from triggering the progression of cancer cells, the modified secretion of molecules can also modulate the behavior of other cellular elements of the TME. Interestingly, CAAs were shown to have immunosuppressive functions during cancer development [45]. Our results suggest that they can also modulate angiogenesis as all of the significantly upregulated adipokines (IL-8, leptin, lipocalin-2, VEGF, and prolactin) were previously described to have pro-angiogenic properties [25–29]. Roy et al. reported that cross-talk between mammary adipocytes and breast-cancer cells promotes tumor aggressiveness and angiogenesis [46]. Thus, the identified molecules may at least partially indicate mechanisms of CAAs' action on cancer cells.

A feature that characterizes the conversion of adipocytes into CAAs is the inhibition of adipogenesis and the differentiation of preadipocytes into mature adipocytes. RACK1 protein is essential in this process due to its roll in inducing Wnt/β-catenin signaling [21]. The levels of both RACK1 and β-catenin were decreased after exposure of adipocytes on CRC cells. Moreover, PPARγ is an adipogenic master regulator that controls the transcriptional activation of the genes involved in lipid uptake and storage [35]. Adipocytes reprogrammed by CRC cells showed decreased expression of *PPARγ,* as well as multiple genes and proteins that this transcription factor controls, which are involved in lipid uptake (*CD36, LDLR, FABPs,* and *FATP1*) and storage (*PLIN and CIDEC*). We also detected a change in the sizes of LDs toward to accumulation of rather small lipid-rich particles in CAAs in comparison to the control adipocytes. The detected alterations might indicate the emerging occurrence of the dedifferentiation process of fat cells in the presence of cancer cells. A similar phenomenon has been previously described with the concomitant loss of lipid content or dispersed LDs [6, 7, 33].

Tumor cells secrete numerous types of factors [47, 48] that induce the transition of adipocytes into CAAs. One of the main mechanisms of this process involves a loss of terminal differentiation markers. A significant reduction in the expression of adipocyte-specific genes, such as PPARγ and FABPs, leads to a dedifferentiated state. Exposing adipocytes to a TME that is rich in TNF-α (tumor necrosis factor α) can downregulate PLIN expression, which mediates lipolysis via pathways that are dependent on ERK1/2 (extracellular signal-regulated kinase) and JNK (c-Jun N-terminal kinase) [49]. Previously, we demonstrated that phosphorylation of Akt (protein kinase B) was substantially decreased in adipocytes co-cultured with CRC cells [11]. Activation of this protein promotes preadipocyte differentiation, while its inactivation inhibits this process [50]. We also found elevated levels of phosphorylated ERK and STAT3 (signal transducer and activator of transcription 3), which may participate in the lipolysis process [11]. The decreased level of pAkt and increased activation of ERK observed in our previous research may represent one of the mechanisms occurring in adipocytes and the inhibition of adipogenesis upon stimulation with CRC cells.

The decreased expression of differentiation factors and the enhanced lipolysis promote the transition of mature adipocytes to cells presenting spindled fibroblast-like phenotype. This process was previously described as an adaptation due to an alteration in the amount of LDs [33]. In light of these changes, some researchers refer to CAAs as adipocyte-derived fibroblasts [51] or consider them to be a source of CAFs, which play an essential role in colorectal metastasis [52]. Although most of the research characterizing CAAs has focused on breast-cancer biology, it is now acknowledged that in all tumors growing in a microenvironment dominated by adipose tissue, adipocytes disappear while fibroblast-like cells accumulate when tumor cells invade the surrounding adipose tissue [10]. In our experimental setting, induced CAAs present more developed F-actin and vimentin networks, however exhibit a non-classical pattern of expression of CAF markers (like *FAP-1, ACTA2, CD90, ITGB1,* and *NES*). This indicates that, in the presence of CRC cells, adipocytes transform only into fibroblast-like cells rather than into a traditional CAF subpopulation. Recent findings have revealed heterogeneity among the CAF population and the existence of multiple functionally distinct subtypes [53]. In light of this, our findings highlight the complexity of the cells' transformation within the TME, which should be explored further in future research.

Aside from phenotypic alterations, the metabolism of CAAs is postulated to switch toward catabolic processes, leading to the release of high-energy metabolites that can be consumed by cancer cells [5]. Adipocytes were demonstrated to support colon-cancer growth and survival by promoting fatty acid oxidation (FAO) in cancer cells [54]. However, there are minimal findings about how cancer cells reprogram the energetic status of adipocytes. Our data clearly show that CAAs exhibit a metabolic shift toward glycolytic ATP production. The detected impairment of the efficiency of the respiratory chain in mitochondria is reflected in the decreased OXPHOS -based ATP production. Several studies have suggested that mitochondria present in the adipose tissue are closely linked to crucial aspects of adipocyte biology, such as adipogenesis and lipid metabolism [55]. Furthermore, a reduced OXPHOS along with the appearance of clusters of mitochondria may be associated with decreased OPA1 and MIEF1 levels in CAAs, and both of these proteins are known to regulate fusion of these organelles. Interestingly, OPA1 was recently shown to be located on LDs in adipocytes, where it serves as a gatekeeper [56]. In a physiological state, mitochondria sustain homeostasis through the processes of fusion and fragmentation. They modify their composition through fragmentation, while damaged organella accumulate dysfunctional components through their fusion. Their morphology is strictly regulated to match the cellular metabolic demands or facilitate its structural rearrangements [57]. Therefore, maintaining mitochondrial quality control through their dynamic rearrangement is essential for proper adipocyte function within the TME.

Several years ago, based on in vivo models or human tumor samples other groups confirmed the existence of CAAs and their tumor-supporting role. CAAs are found at the invasive front of tumors in various cancers, including breast, ovarian, and pancreatic cancers, and their presence correlates with increased tumor progression [5, 10, 58, 59]. Moreover, the complex bidirectional interactions between CAAs and cancer cells contribute to treatment resistance [60]. Our studies identified that CRC cells, apart from morphological changes, induce the metabolic shift concomitant the transformation of adipocytes in their surroundings. Given their significant impact on tumor biology, CAAs and their associated signaling pathways emerge as promising targets for therapeutic strategies to improve cancer treatment outcomes. These aspects of TME biology are especially relevant in the context of obesity, which expands and alters the function of adipose tissue, leading to an enhanced supply of CAAs and further promoting cancer progression.

## Conclusions

We have described the impact of CRC cells on the transformation of human primary adipocytes and their role within the TME. Our results demonstrate that cancer cells induce the appearance of CAAs, which were characterized by a shift in LD profile and the acquisition of fibroblast-like features. Additionally, CAAs secreted more pro-angiogenic adipokines and exhibited altered expression of the proteins involved in lipid storage and metabolism. At the same time, induced CAAs showed decreased mitochondrial respiration levels, accompanied by changes in mitochondrial distribution and dynamics, which may indicate their dysfunction. In conclusion, this work contributes to the understanding of the influence of CRC cells on adipocytes, which may be essential for exploring their role in tumor development and resistance to treatment.

## Data Availability

Data will be made available on request.

## References

[1] Booth A, Magnuson A, Fouts J, Foster M. Adipose tissue, obesity and adipokines: role in cancer promotion. Horm Mol Biol Clin Investig. 2015. 10.1515/hmbci-2014-0037. 25781552 10.1515/hmbci-2014-0037

[2] Richard AJ, White U, Elks CM, Stephens JM. Adipose Tissue: Physiology to Metabolic Dysfunction. Updated 2020 Apr 4. Endotext. 2000.

[3] Coelho P, Almeida J, Prudêncio C, Fernandes R, Soares R. Effect of adipocyte secretome in melanoma progression and vasculogenic mimicry. J Cell Biochem. 2016. 10.1002/jcb.25463. 26666522 10.1002/jcb.25463

[4] Domagalski M, Olszańska J, Pietraszek-Gremplewicz K, Nowak D. The role of adipogenic niche resident cells in colorectal cancer progression in relation to obesity. Obes Rev. 2025;26:e13873. 10.1111/obr.13873. 39763022 10.1111/obr.13873 PMC11884973

[5] Yao H, He S. Multi-faceted role of cancer-associated adipocytes in the tumor microenvironment (Review). Mol Med Rep. 2021. 10.3892/mmr.2021.12506. 34676881 10.3892/mmr.2021.12506 PMC8554381

[6] Suárez–Nájera LE, Chanona–Pérez JJ, Valdivia–Flores A, Marrero–Rodríguez D, Salcedo–Vargas M, García–Ruiz DI, et al. Morphometric study of adipocytes on breast cancer by means of photonic microscopy and image analysis. Microsc Res Tech. 2018. 10.1002/jemt.22972. 29193620 10.1002/jemt.22972

[7] Dirat B, Bochet L, Dabek M, Daviaud D, Dauvillier S, Majed B, et al. Cancer-associated adipocytes exhibit an activated phenotype and contribute to breast cancer invasion. Cancer Res. 2011. 10.1158/0008-5472.CAN-10-3323. 21459803 10.1158/0008-5472.CAN-10-3323

[8] Nieman KM, Romero IL, Van Houten B, Lengyel E. Adipose tissue and adipocytes support tumorigenesis and metastasis. Biochimica et Biophysica Acta (BBA) - Molecular and Cell Biology of Lipids. 2013. 10.1016/j.bbalip.2013.02.010. 23500888 10.1016/j.bbalip.2013.02.010 PMC3742583

[9] Zoico E, Darra E, Rizzatti V, Tebon M, Franceschetti G, Mazzali G, et al. Role of adipose tissue in melanoma cancer microenvironment and progression. Int J Obes. 2018. 10.1038/ijo.2017.218. 10.1038/ijo.2017.218 28883539

[10] Wu Q, Li B, Li Z, Li J, Sun S, Sun S. Cancer-associated adipocytes: key players in breast cancer progression. J Hematol Oncol. 2019. 10.1186/s13045-019-0778-6. 31500658 10.1186/s13045-019-0778-6 PMC6734503

[11] Olszańska J, Pietraszek-Gremplewicz K, Domagalski M, Nowak D. Mutual impact of adipocytes and colorectal cancer cells growing in co-culture conditions. Cell Commun Signal. 2023. 10.1186/s12964-023-01155-8. 37316878 10.1186/s12964-023-01155-8 PMC10265888

[12] Mieczkowska A, Schumacher A, Filipowicz N, Wardowska A, Zieliński M, Madanecki P, et al. Immunophenotyping and transcriptional profiling of in vitro cultured human adipose tissue derived stem cells. Sci Rep. 2018. 10.1038/s41598-018-29477-5. 30054533 10.1038/s41598-018-29477-5 PMC6063933

[13] Zuk PA, Zhu M, Mizuno H, Huang J, Futrell JW, Katz AJ, et al. Multilineage cells from human adipose tissue: implications for cell-based therapies. Tissue Eng. 2001. 10.1089/107632701300062859. 11304456 10.1089/107632701300062859

[14] Pietraszek-Gremplewicz K, Olszańska J, Domagalski M, Simiczyjew A, Kot M, Skoniecka A, et al. Response of primary human adipocytes to fatty acid treatment. J Cell Mol Med. 2025;29:e70622. 10.1111/jcmm.70622. 40418213 10.1111/jcmm.70622 PMC12105583

[15] Wang X, You L, Cui X, Li Y, Wang X, Xu P, et al. Evaluation and optimization of differentiation conditions for human primary brown adipocytes. Sci Rep. 2018;8:5304. 10.1038/s41598-018-23700-z. 29593245 10.1038/s41598-018-23700-z PMC5871774

[16] Laemmli UK. Cleavage of structural proteins during the assembly of the head of bacteriophage T4. Nature. 1970. 10.1038/227680a0. 5432063 10.1038/227680a0

[17] Towbin H, Staehelin T, Gordon J. Electrophoretic transfer of proteins from polyacrylamide gels to nitrocellulose sheets: procedure and some applications. Proc Natl Acad Sci U S A. 1979. 10.1073/pnas.76.9.4350. 388439 10.1073/pnas.76.9.4350 PMC411572

[18] Thacker JS, Yeung DH, Staines WR, Mielke JG. Total protein or high-abundance protein: which offers the best loading control for Western blotting? Anal Biochem. 2016;496:76–8. 10.1016/j.ab.2015.11.022. 26706797 10.1016/j.ab.2015.11.022

[19] Simiczyjew A, Wądzyńska J, Pietraszek-Gremplewicz K, Kot M, Ziętek M, Matkowski R, et al. Melanoma cells induce dedifferentiation and metabolic changes in adipocytes present in the tumor niche. Cell Mol Biol Lett. 2023. 10.1186/s11658-023-00476-3. 37481560 10.1186/s11658-023-00476-3 PMC10363323

[20] An HS, Yoo JW, Jeong JH, Heo M, Hwang SH, Jang HM, et al. Lipocalin-2 promotes acute lung inflammation and oxidative stress by enhancing macrophage iron accumulation. Int J Biol Sci. 2023. 10.7150/ijbs.79915. 36923935 10.7150/ijbs.79915 PMC10008694

[21] Meniailo ME, Malashchenko VV, Shmarov VA, Gazatova ND, Melashchenko OB, Goncharov AG, et al. Interleukin-8 favors pro-inflammatory activity of human monocytes/macrophages. Int Immunopharmacol. 2018. 10.1016/j.intimp.2018.01.036. 29414654 10.1016/j.intimp.2018.01.036

[22] Pérez-Pérez A, Sánchez-Jiménez F, Vilariño-García T, Sánchez-Margalet V. Role of leptin in inflammation and vice versa. Int J Mol Sci. 2020. 10.3390/ijms21165887. 32824322 10.3390/ijms21165887 PMC7460646

[23] Brand JM, Frohn C, Cziupka K, Brockmann C, Kirchner H, Luhm J. Prolactin triggers pro-inflammatory immune responses in peripheral immune cells. Eur Cytokine Netw. 2004. 15319167

[24] Reinders MEJ, Sho M, Izawa A, Wang P, Mukhopadhyay D, Koss KE, et al. Proinflammatory functions of vascular endothelial growth factor in alloimmunity. J Clin Invest. 2003. 10.1172/JCI17712. 14660742 10.1172/JCI17712 PMC281640

[25] Shi J, Wei PK. Interleukin-8: a potent promoter of angiogenesis in gastric cancer. Oncol Lett. 2016. 10.3892/ol.2015.4035. 26893688 10.3892/ol.2015.4035 PMC4734231

[26] Yu F, Fu R, Liu L, Wang X, Wu T, Shen W, et al. Leptin-induced angiogenesis of EA.HY926 endothelial cells via the Akt and Wnt signaling pathways in vitro and in vivo. Front Pharmacol. 2019. 10.3389/fphar.2019.01275. 31736756 10.3389/fphar.2019.01275 PMC6836761

[27] Yang J, McNeish B, Butterfield C, Moses MA. Lipocalin 2 is a novel regulator of angiogenesis in human breast cancer. FASEB J. 2013. 10.1096/fj.12-211730. 22982376 10.1096/fj.12-211730 PMC3528324

[28] Shaw P, Dwivedi SKD, Bhattacharya R, Mukherjee P, Rao G. VEGF signaling: role in angiogenesis and beyond. Biochimica et Biophysica Acta (BBA) - Reviews on Cancer. 2024. 10.1016/j.bbcan.2024.189079. 38280470 10.1016/j.bbcan.2024.189079 PMC12927493

[29] Reuwer AQ, Nowak-Sliwinska P, Mans LA, van der Loos CM, von der Thüsen JH, Twickler MTB, et al. Functional consequences of prolactin signalling in endothelial cells: a potential link with angiogenesis in pathophysiology? J Cell Mol Med. 2012. 10.1111/j.1582-4934.2011.01499.x. 22128761 10.1111/j.1582-4934.2011.01499.x PMC3822974

[30] Kong Q, Gao L, Niu Y, Gongpan P, Xu Y, Li Y, et al. RACK1 is required for adipogenesis. Am J Physiol Cell Physiol. 2016;311:C831–6. 10.1152/ajpcell.00224.2016. 27653985 10.1152/ajpcell.00224.2016

[31] Svitkina TM, Neyfakh AA, Bershadsky AD. Actin cytoskeleton of spread fibroblasts appears to assemble at the cell edges. J Cell Sci. 1986. 10.1242/jcs.82.1.235. 3793781 10.1242/jcs.82.1.235

[32] Wilhelmsson U, Stillemark-Billton P, Borén J, Pekny M. Vimentin is required for normal accumulation of body fat. Biol Chem. 2019. 10.1515/hsz-2019-0170. 30995202 10.1515/hsz-2019-0170

[33] Nieman KM, Kenny HA, Penicka CV, Ladanyi A, Buell-Gutbrod R, Zillhardt MR, et al. Adipocytes promote ovarian cancer metastasis and provide energy for rapid tumor growth. Nat Med. 2011;17:1498–503. 10.1038/nm.2492. 22037646 10.1038/nm.2492 PMC4157349

[34] Kumari RM, Khatri A, Chaudhary R, Choudhary V. Concept of lipid droplet biogenesis. Eur J Cell Biol. 2023. 10.1016/j.ejcb.2023.151362. 37742390 10.1016/j.ejcb.2023.151362 PMC7615795

[35] Auwerx J. PPARγ, the ultimate thrifty gene. Diabetologia. 1999. 10.1007/s001250051268. 10447513 10.1007/s001250051268

[36] Sun Y, Zhai G, Pang Y, Li R, Li Y, Cao Z, et al. PPAR gamma2: the main isoform of PPARγ that positively regulates the expression of the chicken Plin1 gene. J Integr Agric. 2022. 10.1016/S2095-3119(21)63896-0.

[37] Lopes-Coelho F, Gouveia-Fernandes S, Serpa J. Metabolic cooperation between cancer and non-cancerous stromal cells is pivotal in cancer progression. Tumor Biol. 2018. 10.1177/1010428318756203. 10.1177/1010428318756203 29421992

[38] Guinney J, Dienstmann R, Wang X, De Reyniès A, Schlicker A, Soneson C, et al. The consensus molecular subtypes of colorectal cancer. Nat Med. 2015. 10.1038/nm.3967. 26457759 10.1038/nm.3967 PMC4636487

[39] Khaliq AM, Erdogan C, Kurt Z, Turgut SS, Grunvald MW, Rand T, et al. Refining colorectal cancer classification and clinical stratification through a single-cell atlas. Genome Biol. 2022. 10.1186/s13059-022-02677-z. 35538548 10.1186/s13059-022-02677-z PMC9092724

[40] Joanito I, Wirapati P, Zhao N, Nawaz Z, Yeo G, Lee F, et al. Single-cell and bulk transcriptome sequencing identifies two epithelial tumor cell states and refines the consensus molecular classification of colorectal cancer. Nat Genet. 2022. 10.1038/s41588-022-01100-4. 35773407 10.1038/s41588-022-01100-4 PMC9279158

[41] Thanki K, Nicholls ME, Gajjar A, Senagore AJ, Qiu S, Szabo C, et al. Consensus Molecular Subtypes of Colorectal Cancer and their Clinical Implications. Int Biol Biomed J. 2017. PMC5557054 28825047

[42] Kasi A, Handa S, Bhatti S, Umar S, Bansal A, Sun W. Molecular pathogenesis and classification of colorectal carcinoma. Curr Colorectal Cancer Rep. 2020. 10.1007/s11888-020-00458-z. 32905465 10.1007/s11888-020-00458-z PMC7469945

[43] Olszańska J, Pietraszek-Gremplewicz K, Nowak D. Melanoma progression under obesity: focus on adipokines. Cancers. 2021. 10.3390/cancers13092281. 34068679 10.3390/cancers13092281 PMC8126042

[44] Wang YY, Hung AC, Lo S, Yuan SSF. Adipocytokines visfatin and resistin in breast cancer: clinical relevance, biological mechanisms, and therapeutic potential. Cancer Lett. 2021. 10.1016/j.canlet.2020.10.045. 33152400 10.1016/j.canlet.2020.10.045

[45] Wu Q, Li B, Li J, Sun S, Yuan J, Sun S. Cancer-associated adipocytes as immunomodulators in cancer. Biomark Res. 2021. 10.1186/s40364-020-00257-6. 33413697 10.1186/s40364-020-00257-6 PMC7792018

[46] Roy R, Man E, Aldakhlallah R, Gonzalez K, Merritt L, Daisy C, et al. Mammary adipocytes promote breast tumor cell invasion and angiogenesis in the context of menopause and obesity. Biochimica et Biophysica Acta (BBA) - Molecular Basis of Disease. 2024;1870:167325. 10.1016/j.bbadis.2024.167325. 38925485 10.1016/j.bbadis.2024.167325

[47] Tang Y, Zhang W, Sheng T, He X, Xiong X. Overview of the molecular mechanisms contributing to the formation of cancer‑associated adipocytes (Review). Mol Med Rep. 2021. 10.3892/mmr.2021.12408. 34490479 10.3892/mmr.2021.12408 PMC8430316

[48] Wu C, Dong S, Huang R, Chen X. Cancer-associated adipocytes and breast cancer: intertwining in the tumor microenvironment and challenges for cancer therapy. Cancers (Basel). 2023;15:726. 10.3390/cancers15030726. 36765683 10.3390/cancers15030726 PMC9913307

[49] Ryden M, Dicker A, van Harmelen V, Hauner H, Brunnberg M, Perbeck L, et al. Mapping of early signaling events in tumor necrosis factor-alpha -mediated lipolysis in human fat cells. J Biol Chem. 2002;277:1085–91. 10.1074/jbc.M109498200. 11694522 10.1074/jbc.M109498200

[50] Scioli MG, Bielli A, Gentile P, Mazzaglia D, Cervelli V, Orlandi A. The biomolecular basis of adipogenic differentiation of adipose-derived stem cells. Int J Mol Sci. 2014;15:6517–26. 10.3390/ijms15046517. 24743893 10.3390/ijms15046517 PMC4013644

[51] Bochet L, Lehuédé C, Dauvillier S, Wang YY, Dirat B, Laurent V, et al. Adipocyte-derived fibroblasts promote tumor progression and contribute to the desmoplastic reaction in breast cancer. Cancer Res. 2013. 10.1158/0008-5472.CAN-13-0530. 23903958 10.1158/0008-5472.CAN-13-0530

[52] Zhong B, Cheng B, Huang X, Xiao Q, Niu Z, Chen Y feng, et al. Colorectal cancer-associated fibroblasts promote metastasis by up-regulating LRG1 through stromal IL-6/STAT3 signaling. Cell Death Dis. 2022. 10.1038/s41419-021-04461-6 10.1038/s41419-021-04461-6 PMC8688517 34930899

[53] Mathieson L, Koppensteiner L, Dorward DA, O’Connor RA, Akram AR. Cancer-associated fibroblasts expressing fibroblast activation protein and podoplanin in non-small cell lung cancer predict poor clinical outcome. Br J Cancer. 2024;130:1758–69. 10.1038/s41416-024-02671-1. 38582812 10.1038/s41416-024-02671-1 PMC11130154

[54] Wen YA, Xing X, Harris JW, Zaytseva YY, Mitov MI, Napier DL, et al. Adipocytes activate mitochondrial fatty acid oxidation and autophagy to promote tumor growth in colon cancer. Cell Death Dis. 2017;8:1–12. 10.1038/cddis.2017.21. 28151470 10.1038/cddis.2017.21 PMC5386470

[55] Heinonen S, Jokinen R, Rissanen A, Pietiläinen KH. White adipose tissue mitochondrial metabolism in health and in obesity. Obes Rev. 2020. 10.1111/obr.12958. 31777187 10.1111/obr.12958

[56] Pereira RO, Olvera AC, Marti A, Fang S, White JR, Westphal M, et al. OPA1 regulates lipid metabolism and cold-induced browning of white adipose tissue in mice. Diabetes. 2022. 10.2337/db22-0450. 36170659 10.2337/db22-0450 PMC9750944

[57] Song H, Zhang X, Wang J, Wu Y, Xiong T, Shen J, et al. The regulatory role of adipocyte mitochondrial homeostasis in metabolism-related diseases. Front Physiol. 2023. 10.3389/fphys.2023.1261204. 37920803 10.3389/fphys.2023.1261204 PMC10619862

[58] Cai Z, Liang Y, Xing C, Wang H, Hu P, Li J, et al. Cancer-associated adipocytes exhibit distinct phenotypes and facilitate tumor progression in pancreatic cancer. Oncol Rep. 2019;42:2537–49. 10.3892/or.2019.7365. 31638193 10.3892/or.2019.7365 PMC6826327

[59] Cai Q, Yang J, Shen H, Xu W. Cancer-associated adipocytes in the ovarian cancer microenvironment. Am J Cancer Res. 2024;14:3259–79. 10.62347/XZRI9189. 39113876 10.62347/XZRI9189 PMC11301307

[60] Zhao C, Liu S, Gao F, Zou Y, Ren Z, Yu Z. The role of tumor microenvironment reprogramming in primary liver cancer chemotherapy resistance. Front Oncol. 2022. 10.3389/fonc.2022.1008902. 36505831 10.3389/fonc.2022.1008902 PMC9731808

